# Determining Forest Duff Water Content Using a Low-Cost Standing Wave Ratio Sensor

**DOI:** 10.3390/s18020647

**Published:** 2018-02-22

**Authors:** Xiaofei Yan, Yajie Zhao, Qiang Cheng, Xiaoliang Zheng, Yandong Zhao

**Affiliations:** 1 School of Technology, Beijing Forestry University, Beijing 100083, China; yanxf@bjfu.edu.cn (X.Y.); yajie_zh@bjfu.edu.cn (Y.Z.); xiaoliangzh@bjfu.edu.cn (X.Z.); 2 College of Information and Electrical Engineering, China Agricultural University, Beijing 100083, China; chengqiang@cau.edu.cn

**Keywords:** standing wave ratio, forest duff, volumetric water content, compaction

## Abstract

Forest duff (fermentation and humus) water content is an important parameter for fire risk prediction and water resource management. However, accurate determination of forest duff water content is difficult due to its loose structure. This study evaluates the feasibility of a standing wave ratio (SWR) sensor to accurately determine the forest duff water content. The performance of this sensor was tested on fermentation and humus with eight different compaction levels. Meanwhile, a commercialized time domain reflectometry (TDR) was employed for comparison. Calibration results showed that there were strong linear relationships between the volumetric water content (*θ_V_*) and the SWR sensor readings (*V_SWR_*) at different compaction classes for both fermentation and humus samples. The sensor readings of both SWR and TDR underestimated the forest duff water content at low compacted levels, proving that the compaction of forest duff could significantly affect the measurement accuracy of both sensors. Experimental data also showed that the accuracy of the SWR sensor was higher than that of TDR according to the root mean square error (RMSE). Furthermore, low cost is another important advantage of the SWR sensor in comparison with TDR. This low-cost SWR sensor performs well in loose materials and is feasible for evaluating the water content of forest duff. In addition, the results indicate that decomposition of the forest duff should be taken into account for continuous and long-term water content measurement.

## 1. Introduction

The forest floor plays a significant role in forest hydrological processes by affecting water and energy transfer between the sub-canopy atmosphere and the mineral soil [1,2,3]. It can be divided into the surface litter layer (Oi), with freshly fallen plant residue, the sub-surface fermentation layer (Oe), with partially decomposed but still recognizable organic material, and the underlying humus layer (Oa), with well decomposed organic matter [4,5]. Fermentation and humus are collectively known as forest duff. The retention of rainwater by forest duff not only influences the flow into the mineral soil but also provides an important source of water to vegetation [6,7]. Moreover, the moisture status of the forest duff is a critical determinant for fire ignition and spread [8,9], thus understanding the patterns of forest duff water content variation across time and space would be of great benefit to fire risk management and prevention [10]. In forest ecology, forest duff moisture strongly impacts leaf litter decomposition rates, which ultimately affects carbon cycling and microbial activity [11]. Although the importance of forest duff water content has been proved in many different fields, it has received less attention in forest ecosystem research compared with the moisture dynamics in mineral soil. The reason is that it is difficult to keep good contact between the sensor probe and the forest duff due to its loose structure and low density [12,13].

Gravimetry is the most accurate method to determine water content, but the processes of destructive sampling and oven drying are very time-consuming and labor intense. The neutron scattering method can be used to measure water content in porous media with the advantages of being non-destructive and quite accurate [14]. However, this method may result in a hazard for human health [15]. Time domain reflectometry (TDR) is a promising method to determine the forest duff water content in a rapid and accurate way [16]. Börner et al. [12] placed one TDR probe at the interface of the forest floor layer and the mineral soil to measure their average water content, meanwhile the second TDR probe was employed to measure the water content of the mineral soil only. Then the forest floor water content could be calculated by the two-probe configuration. The results showed that this method would underestimate the water content at low moisture contents and overestimate it at high moisture contents. Canone et al. [17] designed a TDR probe with eight blades evenly spaced around the central rod, which would permit more homogeneous spatial distribution of energy and maintain good contact with the surrounding forest floor in the water content measurements. Experimental results indicated that the developed TDR probe performed better than the classic three-rod probe. In addition, a capacitance sensor is an alternative non-destructive technique to continuously measure the forest floor water content [18,19,20].

The natural processes in earthworm-free forests usually keep the forest floor loose and uncompacted. It is well known that forest duff, as a type of loose spongy material, has the characteristics of low density and high porosity, and the lower forest duff tends to have greater compaction and slower moisture loss rates [21]. The distinct spatial variability of forest floor compaction can significantly affect the contact between the sensor probe and the surrounding forest duff, which leads to a large deviation in the water content measurement. However, to the best of our knowledge, no study has been reported examining the effects of the compaction on forest duff water content measurements.

The main objective of this study was to evaluate the performance of a standing wave ratio (SWR) sensor to determine the forest duff water content, and evaluate the accuracy of fermentation and humus moisture measurement at eight different compactions. The behavior of the SWR sensor was compared with the performance of a commercial TDR sensor. Fermentation, humus and mineral soil with different water contents were tested respectively under laboratory conditions.

## 2. Materials and Methods

### 2.1. Water Content Sensors

**Figure 1:**
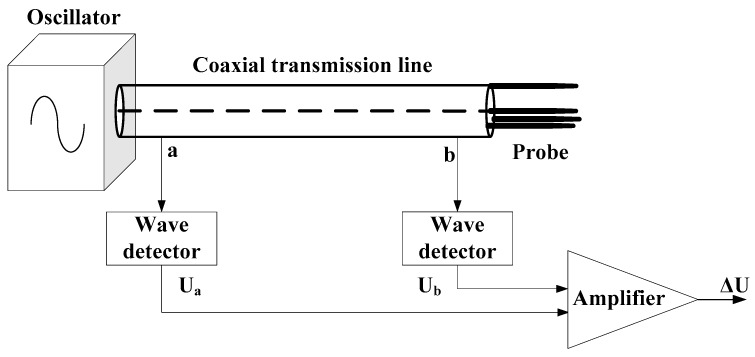
Diagram of the standing wave ratio (SWR) sensor and measurement circuit. U_a_ and U_b_, output voltages of point a and point b on the coaxial transmission line, respectively; Δ*U*, differential output voltage of point a and point b.

**Figure 2:**
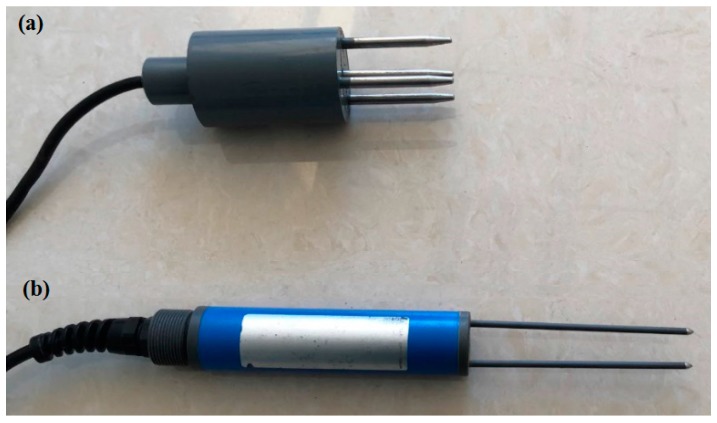
Photograph of the probes used: (**a**) the SWR probe; (**b**) the commercial time domain reflectometry (TDR) probe.

The standing wave ratio (SWR) sensor, also known as an impedance sensor or amplitude domain reflectometry, consisted of a 100 MHz sinusoidal oscillator, a coaxial transmission line, and a stainless steel sensing probe (Figure 1). The high frequency measurement (higher than 30 MHz) can minimize the measurement uncertainty induced by the solutes in water [22,23]. The electromagnetic wave provided by the sinusoidal oscillator spreads along the coaxial transmission line into the probe, and if the impedance of the probe is different from that of the transmission line, a proportion of the incident wave would be reflected back along the line to the oscillator. As a result of the reflected wave interfering with the incident wave, a voltage standing wave would be set up on the transmission line. Therefore, voltage difference (*ΔU*) can be measured at both ends of the transmission line, and it can be expressed as a function of the probe impedance [23]:

(1) $$\Delta U=2A\frac{Z_{P}-Z_{L}}{Z_{P}+Z_{L}}$$

where *A* is the electromagnetic wave amplitude (V), *Z_P_* is the probe impedance (Ω), and *Z_L_* is the transmission line impedance (Ω), which is dependent on its physical dimensions and dielectric constant of the insulating material. *Z_L_* is equal to 50 Ω in this research. Moreover, *Z_P_* is determined by the dielectric constant of the surrounding material of the probe because its geometry has been immobilized. In other words, *Z_P_* is decided by the water content when the fixed probe is inserted into some porous medium. Thus, we can quantify the forest floor or mineral soil moisture by measuring the voltage difference (*ΔU*) of the transmission line. As shown in Figure 2a, the length and diameter of the SWR sensor developed in this work were 16 cm and 5 cm, respectively. It haf four stainless steel rods of approximately 5.8 mm diameter and 5 cm length. Three rods were evenly disposed along a cylinder and one in the center. Furthermore, the same distance between the central rod and the other three rods was approximately 19 mm from center to center. All rods were inserted in a brass support to form a coaxial probe.

TDR can determine the dielectric constant of materials surrounding the probe to measure the volumetric water content. The electromagnetic wave travels down a coaxial cable to the TDR probe. Part of the wave would be reflected in the joint of the cable and the probe due to the impedance difference, and the remainder of the wave would propagate through the probe and return to the source at the end of the probe. The round-trip time of the wave is described as [24]:

(2) $$t=\frac{2L\varepsilon^{0.5}}{c}$$

where *t* is the round-trip time (s); *L* is the TDR probe length (m); *ε* is the dielectric constant of the medium, and *c* is the velocity of electromagnetic wave in free space (3 × 10^8^ m s^−^^1^). Due to the fact that the dielectric constant of free water (≈80) is significantly greater than that of dry solid wood (2~5), dry soil (3~8), and air (≈1), the changes in dielectric constants of forest floor and mineral soil may be predominantly attributed to water content [25,26,27]. Consequently, the round-trip time would be proportional to the water content of the material surrounding the probe [28]. In this work, a portable TDR probe (TRIME-PICO 64, IMKO, Ettlingen, Germany) equipped with a data-reader (HD2, IMKO, Germany) was used to measure the forest floor and mineral soil moisture. As shown in Figure 2b, the TDR probe has two stainless steel rods of 3 mm diameter and 114 mm length. The space of the rod is 20 mm from center to center. Water content measurements were recorded by the data-reader in the unit of volumetric water content (cm^3^ water × cm^−^^3^ forest floor or mineral soil).

### 2.2. Potential Uncertainty Due to Temperature Effects on Dielectric Sensor Readings

Temperature effects on forest duff water content measurements using dielectric sensors stemming from the temperature-dependent response of the sensor circuit itself and the temperature dependence of the dielectric properties of the medium (e.g., the water in the forest duff). The former has been characterized by Saito et al. (2009) [29]. In this study, a potential uncertainty due to temperature effects on duff permittivity was considered by combining a permittivity model developed for porous materials [30]:

(3) $$\varepsilon^{\alpha }=\varphi_{l}\varepsilon_{l}{\left(T\right)}^{\alpha }+\varphi_{s}\varepsilon_{s}{}^{\alpha }+\varphi_{a}\varepsilon_{a}{}^{\alpha }$$

(4) $$\varphi_{l}+\varphi_{s}+\varphi_{a}\;=\;1$$

with a temperature-dependent equation describing the permittivity of free water [31]:

(5) $$\varepsilon_{l}\left(T\right)=78.54[1-4.58\times \left(T-25\right)+1.19\times 10^{-5}{\left(T-25\right)}^{2}-2.80\times 10^{-8}{\left(T-25\right)}^{3}]$$

Equations (3)–(5) were coupled to estimate the potential uncertainty caused by the temperature dependence of the dielectric properties of the medium. In these equations, *ε* is the permittivity, *ϕ* is of the volumetric fraction of each component in the forest duff, and the associated subscripts *l*, *s* and *a* refer to water, solid component and air, respectively. Besides, *α* is an empirical calibration exponent [32] and can be determined by a method proposed by Roth et al. (1990) [33]. In this study, we found that *α* = 0.85 fitted well to Equation (5) with R^2^ = 0.963.

### 2.3. Field Sampling

The samples of fermentation, humus and mineral soil were collected from a forest ecosystem in Jiufeng National Forest Park (39°54′ N, 116°28′ E), Beijing, China. This park is the property of Beijing Forestry University and is used for teaching and scientific research. The climate is a typical warm-temperate continental climate with a mean annual temperature of 11.6 °C and mean annual precipitation of 600 mm, of which 80% falls during the period from June to September [34,35]. The sampling site is a homogeneous forest where the dominant species is *Quercus variabilis* and the forest floor is approximately 12 cm thick (the Oi, Oe and Oa layers are about 2, 4 and 6 cm thick, respectively). The boundary of the litter, fermentation, humus and mineral soil are flat and abrupt. For sampling of the fermentation, humus and the surface mineral soil (0~10 cm), the horizon to be sampled was first uncovered with the help of a shovel and knife. All of the samples were collected vertically.

### 2.4. Experimental Procedure

The water contents of fermentation and humus were tested using SWR and TDR at eight different compactions. The samples of fermentation and humus were oven-dried at 65 °C for 48 h, and mineral soil was oven-dried at 105 °C for 24 h. In order to prepare samples with different compactions, a procedure similar to the Proctor compaction test described by Ayers and Perumpral (1982) was conducted [36]. A polyvinyl chloride (PVC) cylindrical mold (diameter = 15 cm, height = 21 cm) was used to pack the sample with a PVC rod (diameter = 5 cm, length = 48 cm, weight = 1 kg) as the drop hammer. Densification was carried out by dropping the hammer at a constant height of 20 cm when sample was placed into the mold in layers. The eight different compaction levels of fermentation and humus samples were achieved by zero, one, three, six, eight, 10, 15 and 20 blows per layer, respectively.

**Figure 3:**
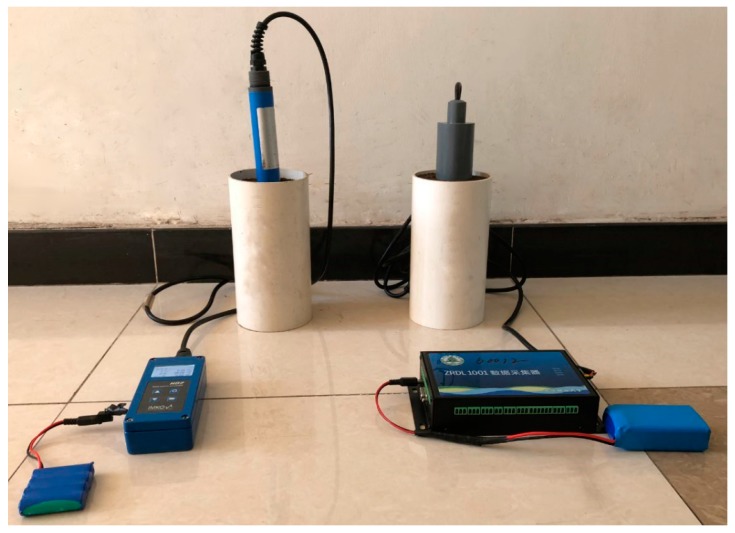
Photograph of the experimental setup for water content measurement by TDR and SWR.

The experimental procedure was performed as follows. First the samples were oven-dried and the empty mold was weighed. Then the mold was packed by placing samples in layers with a predetermined number of blows per layer. The mold with sample was weighed and the volume of the packed sample was calculated to determine the volumetric water content (*θ_V_*). After that, the compaction of the prepared sample was measured using a hand-held digital SC-900 Soil Compaction Meter (Spectrum Technologies, Inc., Plainfield, IL, USA). Moreover, as shown in Figure 3, the water content of this sample was measured by both TDR and SWR sensors. All tests were repeated twice on the prepared sample at the same volumetric water content and compaction. Furthermore, the compaction and water content measurements were conducted in the same way on samples prepared at seven other compactions keeping the same gravimetric water content (*θ_G_*). After the twenty-four tests at eight different compactions were finished, the gravimetric water content of the sample was increased by adding a certain amount of deionized water (100 g) in a fine mist form and mixing thoroughly. Then the same experimental procedure to test the compaction and water content were conducted at the eight different compactions. After that, deionized water was added at 100 g intervals until the sample reached saturation, and the measurements of compaction and water content were carried out as mentioned above.

## 3. Results and Discussion

### 3.1. Effect of Compaction on Sensor Readings for Water Content Measurement

**Figure 4:**
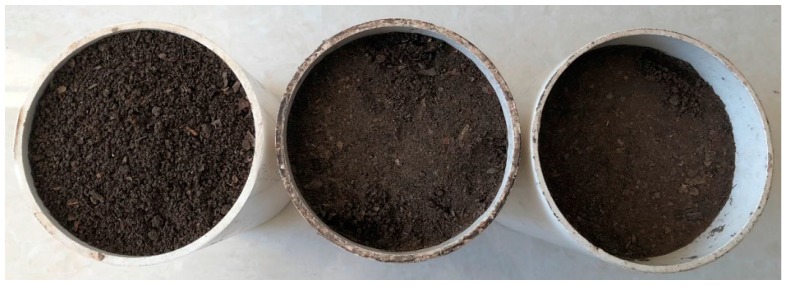
The same quality of humus samples with three compaction classes. The left, middle and right samples were incompact with zero blows per layer, slightly compacted with three blows per layer and strongly compacted with 15 blows per layer, respectively.

In order to keep better linear relationships between volumetric water content determined gravimetrically (*θ_V_*) and SWR output voltage (*V_SWR_*), the eight different compaction levels of fermentation and humus samples were divided into three compaction classes: incompact, slightly compacted and strongly compacted. The photograph in Figure 4 presented the same quality of humus samples prepared with incompact, slightly and strongly compacted classes. The range of compaction measured by SC-900 and the bulk density of fermentation and humus are shown in Table 1.

**Figure 5:**
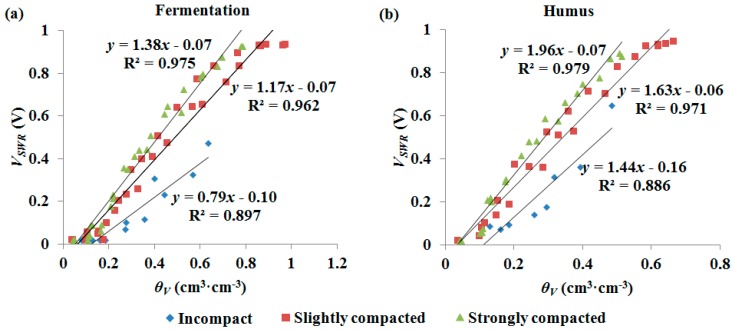
Volumetric water content determined gravimetrically (*θ_V_*) vs. SWR output voltage (*V_SWR_*) for the samples of fermentation (**a**) and humus (**b**) in different compaction classes.

**Figure 6:**
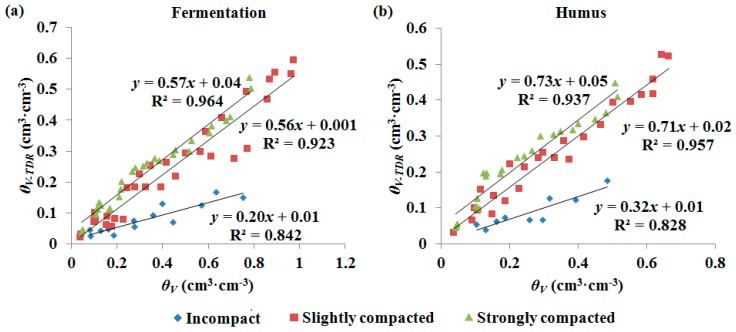
Volumetric water content determined gravimetrically (*θ_V_*) vs. TDR measured volumetric water content (*θ_V-TDR_*) for the samples of fermentation (**a**) and humus (**b**) in different compaction classes.

Strong linear relationships between *θ_V_* and *V_SWR_* in the three compaction classes for both the fermentation and humus samples were found. As shown in Figure 5, the variation of compaction resulted in noticeable changes in calibration curves, and the responses of *V_SWR_* to compaction variation showed similar patterns for fermentation and humus in water content measurements. The more the sample was compacted, the lower the volumetric water content was determined at the same reading of SWR. Furthermore, similar results were also observed using TDR to measure the water content of the fermentation and humus in the above compaction classes (Figure 6). Reports in some of the literature indicated that the compaction of mineral soil is commonly assessed through soil bulk density and is also influenced by gravimetric soil water content, and compaction only slightly affects the water content measurements in mineral soil using dielectric sensors such as SWR and TDR, because these sensors, used to determine volumetric water content, include the information on the bulk density and gravimetric water content of mineral soils [24,37]. However, our experimental results demonstrated that there is an obvious dependence on compaction for volumetric water content measurements in fermentation and humus. Compared to mineral soil, fermentation and humus have a loose spongy structure with higher porosity and greater variation in bulk density, so compaction is a critical factor and should not be neglected when measuring forest duff water content.

The root mean square error (RMSE) of the regression values was calculated to evaluate calibration accuracy (Table 2). When the samples’ water content was determined using the SWR sensor, the RMSEs of the incompact samples (0.058 and 0.041 cm^3^·cm^−^^3^ for fermentation and humus, respectively) were distinctly higher than those of the strongly compacted samples (0.036 and 0.025 cm^3^·cm^−^^3^ for fermentation and humus, respectively). Similarly, moisture measurements of the incompact samples using TDR also showed larger errors (RMSEs of 0.083 and 0.050 cm^3^·cm^−^^3^ for fermentation and humus, respectively) than those of the strongly compacted samples (0.043 and 0.039 cm^3^·cm^−^^3^ for fermentation and humus, respectively). The results indicated that the performance of both SWR and TDR was better in measuring the water content of fermentation and humus with higher compaction. This might be due to the fact that loose materials such as fermentation and humus with lower compaction could not be prepared as uniformly as the samples with higher compaction, and the sensor probes are inserted in large pore spaces randomly.

### 3.2. Comparison of Calibration Curves between Fermentation, Humus and Soil

**Figure 7:**
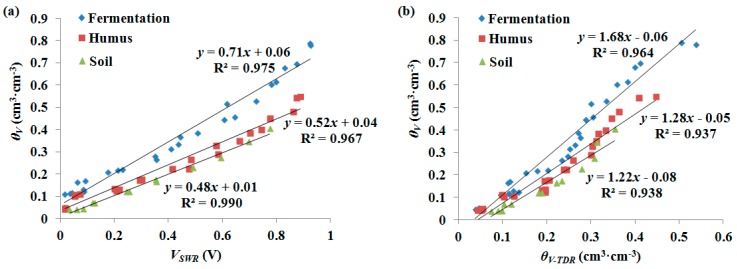
Comparison of volumetric water content determined gravimetrically (*θ_V_*) vs. (**a**) SWR output voltage (*V_SWR_*) and (**b**) TDR measured volumetric water content (*θ_V-TDR_*) between fermentation, humus and soil.

Figure 7 shows the linear relationships between the *θ_V_* and the sensor readings of fermentation, humus and mineral soil when the samples were strongly compacted. For both the SWR and TDR sensors, the calibration curve of fermentation was significantly different from that of humus and soil. This is because the fermentation is partially decomposed and remains of a fibrous nature, such as plant roots, leaves and twigs, which is more easily moistened or dried than the colloidal humus and soil. Moreover, the calibration curves of humus and soil were close and the volumetric water content of humus was slightly higher than soil at the same sensor reading. This is due to their similar textural compositions and higher organic matter in humus than soil. Therefore, it should be noticed that both SWR and TDR are susceptible to the decomposition of organic matter. The structure of the forest floor changes with decomposition over time, which can lead to an overestimation of the water content measurement in situ. Thus, sensors should be recalibrated after installation at a certain time interval according to the decomposition rate of organic matter.

### 3.3. Evaluating the Performance of the SWR Sensor

RMSE values were used to evaluate the performances of the SWR and TDR in determining the water content of fermentation and humus (Table 2). Comparing with *θ_V_*, the RMSEs of the regression values were 0.058 and 0.041 cm^3^·cm^−^^3^ for SWR, and 0.083 and 0.050 cm^3^·cm^−^^3^ for TDR in the incompact fermentation and humus, respectively. In addition, the RMSEs of the SWR were also lower than those of the TDR in the slightly and strongly compacted samples. Compared to the TDR data, the SWR data showed slightly less scatter and greater *R*^2^ values (Figure 7). Consequently, the measurement accuracy of the SWR sensor was higher than that of the TDR sensor. This might be due to the geometry of the SWR sensor, which permits better contact with the surrounding porous material and a more homogeneous spatial distribution of the electromagnetic field in the space surrounded by the probes.

As shown in Figure 5, when the water content of fermentation or humus was close to saturation, the accuracy of the SWR sensor began to decrease. This is because impedance response of the SWR sensor is changed from capacitive to inductive when the water content is going to saturate, and the output of the SWR sensor is no longer raised with the increase of sample water content. However, this can be improved by adjusting the characteristic impedance to increase the sensitivity at high-level water contents. In comparison, the sensitivity of TDR sensor at high-level water contents of fermentation and humus were also slightly reduced, since the instrument was developed for mineral soil water content measurement.

The TDR sensor has been widely used for water content measurement in forest floors and soils. However, the commercial TDR instruments are expensive, and their prices are generally higher than $2000. Forest floor water content usually has a notable spatial and temporal variability, thus many moisture sensors are often required to obtain reliable data. Comparing with TDR, the greatest advantage of the SWR is its low cost. The price of our SWR sensor is no more than $100. This means that a large number of sensors can be deployed to estimate the pattern of water content in a field with great spatial variability. Consequently, the SWR sensor is effective at measuring fermentation and humus moisture over a wide range of water content, and it can provide a potential for continuous, remote measurements with very limited maintenance.

## 4. Conclusions

This study evaluated a moisture sensor based on the principle of standing wave ratio to determine forest duff water content under laboratory conditions. We experimentally investigated the influence of compaction on the calibration procedure of water content measurements in fermentation and humus. Besides, the performance of the SWR sensor was tested in comparison with that of the TDR sensor. Based on the experimental results, the following conclusions can be drawn:

(1) Our study confirmed that the factor of compaction can strongly influence forest duff water content measurements using dielectric permittivity sensors because forest duff is generally looser than mineral soil. Both the SWR and TDR were sensitive to changes in the compaction of fermentation and humus, and better measurement accuracy was found in determining the water content of strongly compacted samples.

(2) Significant differences between the calibration curves of fermentation and humus suggested that decomposition over time within the forest duff could affect the relationship between the sensor readings and volumetric water contents of the forest duff. Therefore, frequent recalibration was necessary after water content sensors were installed in situ.

(3) Both the SWR or TDR had good accuracy for determining the forest duff water content. According to the RMSE values, the performance of the SWR sensor was slightly better than that of the TDR sensor. One disadvantage of the SWR used in the forest duff moisture measurement was that the sensor accuracy decreases when the samples of fermentation or humus were close to saturation. However, the SWR sensor is low cost, so a large number of sensors can be installed in situ at a much lower cost than using TDR. Therefore, compared with TDR, the SWR sensor was better suited to the continuous forest duff water content measurements.

(4) Future studies should focus on further improving SWR accuracy in forest duff moisture measurements. Considering that forest duff decomposes and recalibration is needed for long-term water content measurements, evaluating the decomposition degree of forest duff should be taken into account. In addition, forest duff water content, which is generally estimated by meteorological factors, plays an important role in determining the ignition probability and spread of forest fire. Measuring forest duff water content directly and accurately can improve the level of forest fire management significantly.

## Figures and Tables

**Table 1:** Classification of blows per layer in the Proctor compaction test used for the characterization of sample compaction.

Sample Type	Blows per Layer	Compaction Measured by SC-900 (kPa)	Bulk Density (g/cm^3^)	Compaction Classes
Fermentation	0, 1	<100	0.54~0.63	Incompact
	3, 6, 8	105~455	0.65~0.86	Slightly compacted
	10, 15, 20	560~1260	0.89~1.12	Strongly compacted
Humus	0, 1	<100	0.78~0.84	Incompact
	3, 6, 8	105~490	0.87~0.96	Slightly compacted
	10, 15, 20	560~1435	1.01~1.28	Strongly compacted

**Table 2:** Root mean square error (RMSE) of volumetric water content measurements using SWR and TDR in fermentation and humus with three different compaction classes.

Sample Type	Compaction Classes	RMSE (cm^3^·cm^−^^3^)	
		SWR	TDR
Fermentation	Incompact	0.058	0.083
	Slightly compacted	0.057	0.081
	Strongly compacted	0.036	0.043
Humus	Incompact	0.041	0.050
	Slightly compacted	0.033	0.042
	Strongly compacted	0.025	0.039
